# Real-World Effectiveness of Egg-Based Inactivated Influenza Vaccines Against Influenza Among Children Aged 6–59 Months During the 2025/26 Season in Shenzhen Area, China: A Retrospective Cohort Study

**DOI:** 10.3390/vaccines14090762

**Published:** 2026-08-31

**Authors:** Hui-Ling Tan, Wu Li, Yu Zeng, Rui-Kun Zeng, Jia-Xin Zhong, Hou-Ming Wei

**Affiliations:** 1 Longgang District Center for Disease Control and Prevention, Shenzhen 518172, China; 2 School of Public Health, Guangdong Medical University, Dongguan 523808, China

**Keywords:** influenza, inactive vaccine, effectiveness, children, retrospective cohort study

## Abstract

**Background**: Young children are at high risk of influenza and its complications, yet evidence on the real-world effectiveness of egg-based inactivated influenza vaccines in children aged 6–59 months remains limited during antigenically drifted seasons. **Methods**: We conducted a population-based retrospective cohort study using linked immunization and influenza surveillance records from 4 August 2025 to 8 February 2026. Vaccinated and unvaccinated children aged 6–59 months were matched 1:1 using propensity scores based on demographic characteristics, routine and non-routine vaccination history, prior seasonal influenza vaccination (SIV), and prior influenza infection. For vaccinated children, the index date was defined as the date of the last SIV received during the study period; matched unvaccinated children were assigned the corresponding index date. Follow-up began 14 days later and continued until influenza onset or 8 February 2026. Adjusted vaccine effectiveness (aVE) and 95% confidence intervals (CIs) were estimated using Cox proportional hazards models. Sensitivity analyses included a negative control outcome analysis using varicella infection, a multivariable logistic regression analysis with influenza infection as the outcome, and a time-dependent exposure Cox regression analysis in the full eligible cohort. **Results**: A total of 26,444 children were included in the final cohort (13,239 vaccinated and 13,205 unvaccinated). Influenza incidence rates were 28.70 and 40.21 per 100 person-years in vaccinated and unvaccinated groups, respectively. The aVE was 28.82% (95% CI: 23.07–34.14%). aVE was generally consistent across most subgroups, but was lower in children vaccinated in the previous season than in those without prior-season vaccination (20.41% vs. 34.28%; *p* for interaction = 0.016). Sensitivity analyses yielded comparable VE estimates. **Conclusions**: Egg-based inactivated influenza vaccination conferred moderate real-world protection against influenza in children aged 6–59 months during the 2025/26 season. Continued monitoring of vaccine effectiveness is needed to inform vaccination strategies in young children.

## 1. Introduction

Seasonal influenza is an acute respiratory infection caused by influenza viruses and remains a major global public health concern. Each year, seasonal epidemics are estimated to infect approximately 10% of the world’s population [1,2], with infection rates nearly twice as high among children under 5 years of age [1,3]. In 2018, influenza was estimated to be associated with approximately 870,000 hospitalizations and 34,800 deaths from acute lower respiratory infections among children younger than 5 years worldwide [4]. This substantial disease burden places considerable impact on healthcare systems and socio-economic development.

Seasonal influenza vaccination (SIV) is one of the most cost-effective strategies for reducing influenza-related disease burden. Children aged 6–59 months are recommended as a priority group for annual vaccination [2,5]. However, influenza vaccination coverage in this population varies substantially across regions, ranging from 2.9% [6] to 57.5% [7,8], owing to differences in vaccination policies, accessibility, and parental perceptions of vaccine safety and effectiveness.

Previous studies have shown that SIV can reduce the risk of influenza infection [9,10] and severe outcomes in young children [11]. However, vaccine effectiveness (VE) varies considerably across seasons and may be influenced by the antigenic match between vaccine and circulating strains [12], number of doses received [13], prior vaccination history [14,15], vaccine type [16], and waning immunity [17,18]. During the 2025/26 Northern Hemisphere influenza season, a drifted A(H3N2) variant, J.2.4.1 (also referred to as subclade K), emerged rapidly and became predominant in multiple regions. This subclade harbors multiple amino acid substitutions in the hemagglutinin protein [19], which may contribute to immune escape and reduced vaccine effectiveness [20,21]. Although preliminary evidence from some regions suggests moderate protection [22,23,24], additional real-world evidence from different geographic settings and populations is needed to assess the consistency and generalizability of these findings.

In China, all influenza vaccines licensed are egg-based products at present [5]. Egg-adaptive mutations arising during vaccine production may further compromise vaccine effectiveness [25,26]. However, real-world evidence on the effectiveness of egg-based influenza vaccines remains limited in seasons characterized by substantial antigenic drift. Therefore, we estimated the real-world effectiveness of egg-based inactivated influenza vaccines against influenza in children aged 6–59 months during the 2025/26 influenza season in Shenzhen, China, to inform vaccination strategies under drifted epidemic conditions.

## 2. Methods

### 2.1. Study Design and Data Sources

This retrospective cohort study was conducted in Longgang District, Shenzhen, China. The study population comprised children aged 6–59 months who were registered for kindergarten or childcare enrollment in August 2025. Individual-level data, including demographic characteristics (date of birth and sex) and vaccination records, were extracted from the Shenzhen Immunization Program Information System, which routinely captures domestic vaccination records for children registered for kindergarten or childcare enrollment in Shenzhen. Vaccination records included National Immunization Program (NIP) vaccines and records of non-NIP vaccinations, such as SIV, pneumococcal vaccination, enterovirus 71 (EV71) vaccination, and varicella vaccination.

Information on influenza cases, including date of onset, case classification, and virus type, was extracted from the National Notifiable Infectious Disease Surveillance System, which records influenza cases reported by healthcare institutions in China. Influenza cases were defined according to the Protocol for Diagnosis and Treatment of Influenza (2025 edition) [27]. Clinically diagnosed cases were defined as individuals with epidemiological exposure (e.g., close contact with suspected or confirmed influenza cases within 7 days before illness onset, participation in clustered outbreaks, or evidence of transmission) and influenza-like illness, with other causes excluded. Laboratory-confirmed cases were defined as individuals with influenza-like illness and a positive influenza antigen or nucleic acid test result. The surveillance data did not provide sufficiently complete and standardized information on the specific diagnostic assay for each case; therefore, laboratory-confirmed cases were analyzed as a single category without further stratification by testing method. Based on laboratory findings, confirmed cases were further classified as influenza A, influenza B, or co-infection. Subtype-specific analyses were not performed due to incomplete subtype information in the surveillance database. No additional questionnaires were administered to children or their parents. After data cleaning, deduplication, and record linkage, an integrated analytic dataset containing vaccination histories and influenza outcomes was established.

### 2.2. Study Population and Cohort Construction

**Figure 1:**
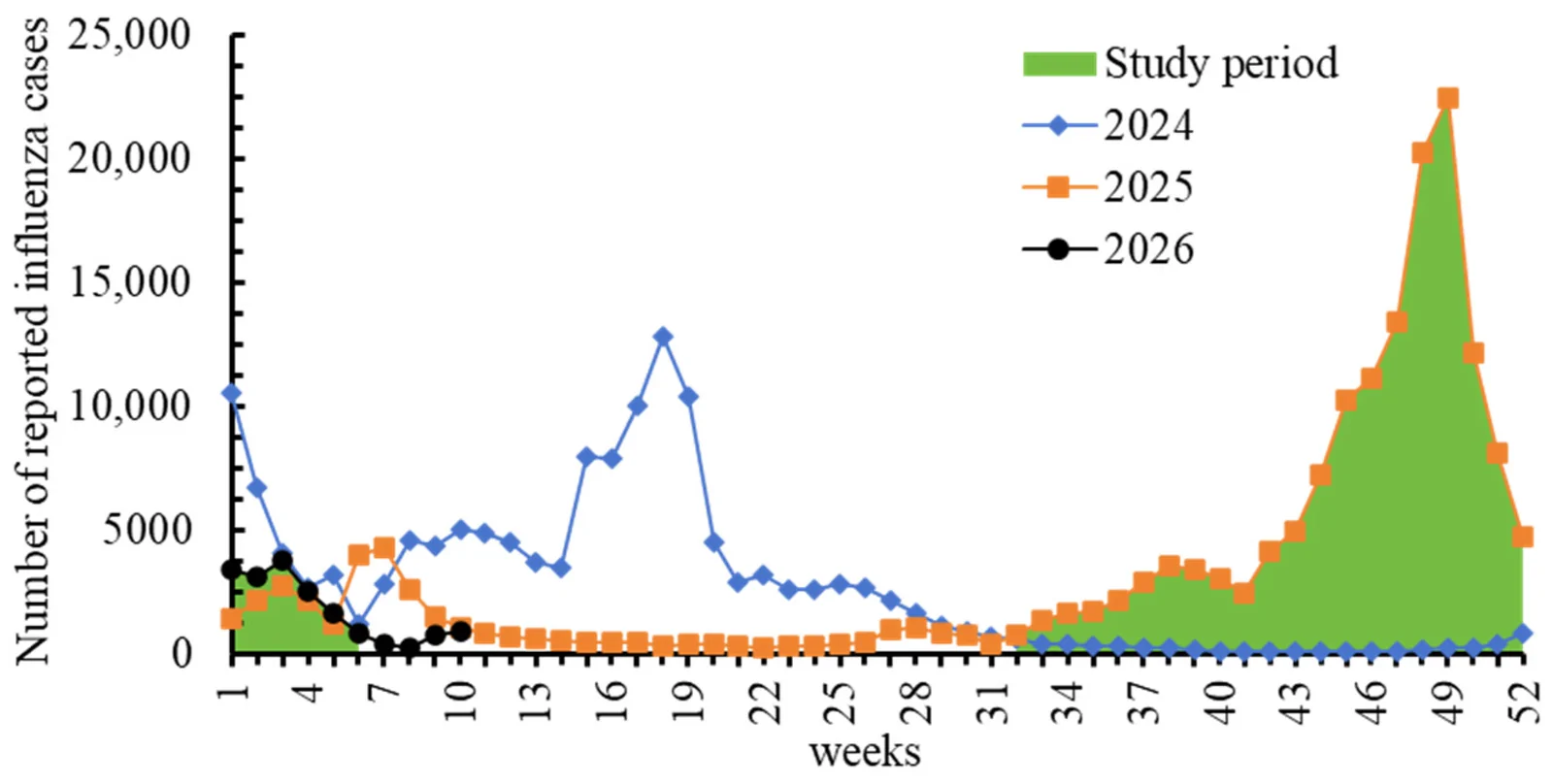
Weekly trends in reported influenza cases in Longgang District, Shenzhen, from week 1 of 2024 to week 10 of 2026.

The study period was defined as the 2025/26 influenza season, from 4 August 2025 (week 32) to 8 February 2026 (week 6), based on the local temporal pattern of reported influenza activity (Figure 1). A total of 108,774 children were initially registered. After excluding 565 children who were outside the eligible age range (6–59 months) and 1017 children who had received SIV or developed influenza between 5 May 2025 (week 19) and 3 August 2025 (week 31), to reduce potential bias related to recent vaccination or infection before the start of the study season, 107,192 children were included in the initial cohort, comprising 14,291 vaccinated and 92,901 unvaccinated children.

To minimize confounding, propensity scores were estimated using a logistic regression model including age, sex, completion of the full NIP schedule, delayed receipt of any age-eligible NIP vaccine dose, history of EV71 vaccination, pneumococcal vaccination, varicella vaccination, SIV in the previous season, and influenza infection in the previous season. One-to-one nearest-neighbor matching without replacement was then performed using a caliper width of 0.2 standard deviations of the logit of the propensity score.

After matching, 14,291 vaccinated children were matched to 14,291 unvaccinated children. For the vaccinated group, the index date was defined as the date of the final SIV dose received during the study period. For matched unvaccinated children, the index date was assigned as the corresponding index date of their matched vaccinated counterparts within the same matched pair, in order to align calendar time and epidemic phase between groups. To allow time for the development of vaccine-induced immunity, follow-up began 14 days after the index date. Children who developed influenza within 14 days after the index date were excluded (*n* = 2138, 1052 in the vaccinated group and 1086 in the unvaccinated group). The final analytic cohort therefore included 26,444 children, of whom 13,239 were vaccinated and 13,205 were unvaccinated.

**Figure 2:**
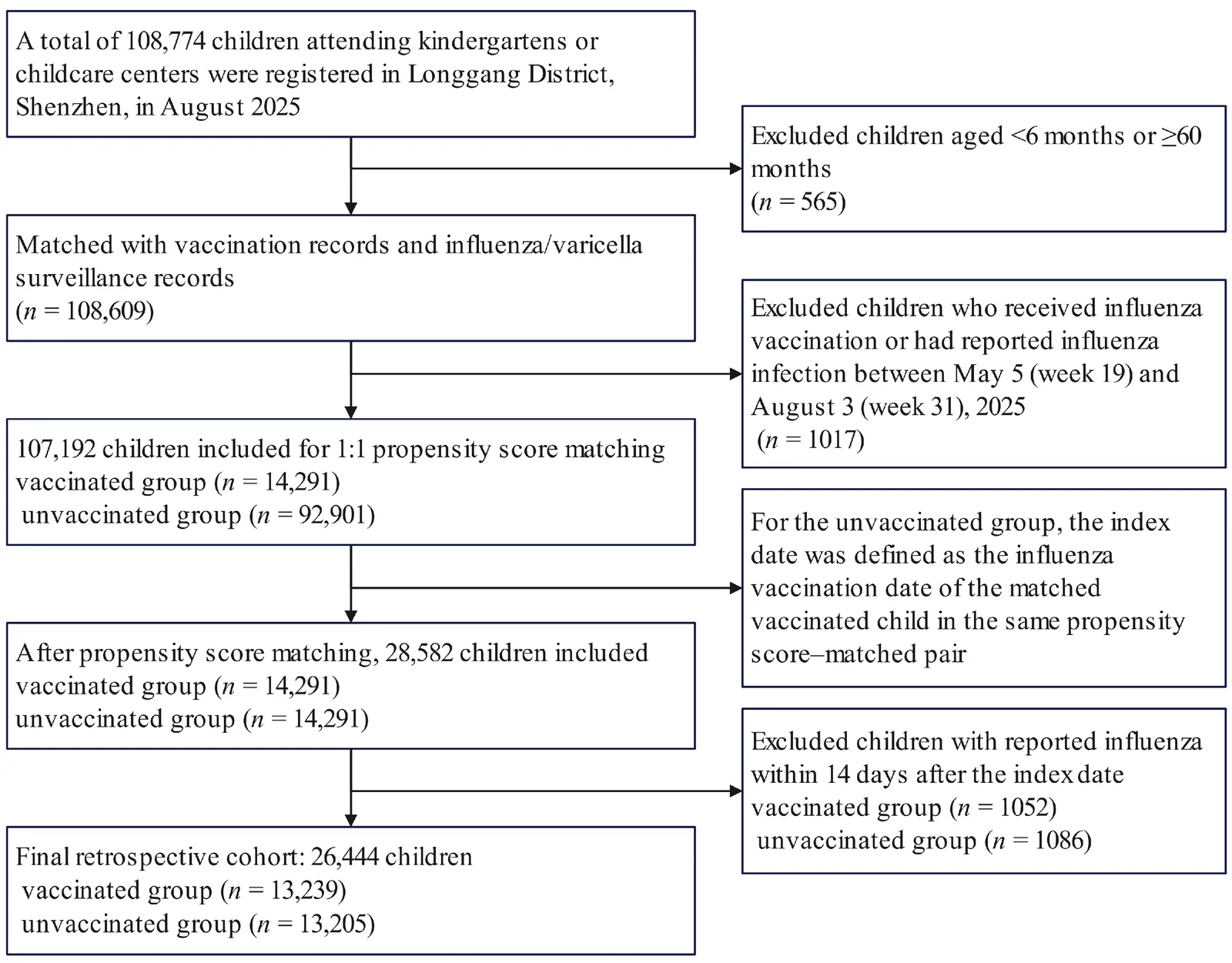
Flowchart of the inclusion and exclusion process for study participants.

Among the vaccinated children, 99.3% received egg-based split-virion inactivated quadrivalent influenza vaccine, whereas 0.7% received egg-based split-virion inactivated trivalent influenza vaccine. Because these post-matching exclusions were applied at the individual level, the final analytic cohort no longer retained an exact 1:1 matched structure. Follow-up began 14 days after the index date and continued until influenza onset or the end of follow-up (8 February 2026), whichever came first. The process of participant selection, exclusion, matching, and construction of the final analytic cohort is shown in Figure 2.

### 2.3. Variable Definitions

Covariates were defined on the basis of vaccination records as of 4 August 2025. Completion of the full NIP schedule was defined as receipt of all recommended NIP vaccine doses, including eligible substitute non-NIP vaccines where applicable. Delayed receipt of any age-eligible NIP vaccine dose was defined as failure to receive a recommended NIP vaccine dose or its eligible substitute after reaching the scheduled age for vaccination. History of EV71, pneumococcal, and varicella vaccination was defined as receipt of at least one dose of the corresponding vaccine before 4 August 2025. Pneumococcal vaccination included both the 13-valent pneumococcal conjugate vaccine (PCV13) and the 23-valent pneumococcal polysaccharide vaccine (PPSV23). History of SIV and influenza infection was defined on basis of records from the previous influenza season (August 2024 to April 2025).

### 2.4. Statistical Analysis

Baseline characteristics were summarized using counts and percentages. Covariate balance between vaccinated and unvaccinated groups before and after matching was assessed using absolute standardized differences (ASDs), with values < 0.1 indicating adequate balance. Person-time was calculated from 14 days after the index date to the date of influenza onset or the end of follow-up, whichever occurred first. Incidence rates were expressed as the number of influenza cases per 100 person-years.

The proportional hazards assumption was assessed using Schoenfeld residuals, and no major violation was detected. Multivariable Cox proportional hazards regression models were used to estimate adjusted hazard ratios (aHRs) and 95% confidence intervals (CIs). Because exclusions within 14 days after the index date were applied at the individual level after matching, the exact matched-pair structure was not fully preserved in the final analytic cohort. Therefore, all baseline covariates included in the propensity score model were re-entered into the multivariable Cox models to further control for residual confounding. Adjusted vaccine effectiveness (aVE) was calculated as (1 − aHR) × 100% when aHR ≤ 1, and as ([1/aHR] − 1) × 100% when aHR > 1 [28].

Subgroup analyses were conducted according to age, sex, vaccination history, and other covariates, and interaction effects were assessed. Sensitivity analyses were performed to evaluate the robustness of the findings. First, a negative control outcome analysis using varicella infection was conducted to detect potential residual confounding. Varicella infection was selected as a negative control outcome because it is not expected to be biologically affected by influenza vaccination, while being captured within the same surveillance framework. Second, a multivariable logistic regression model was used as an alternative analytical approach, with influenza infection during follow-up as the dependent variable and vaccination status as the primary independent variable, adjusting for the same covariates as in the main Cox model. Adjusted odds ratios (aORs) and 95% CIs were estimated, aVE was calculated as (1 − aOR) × 100% when aOR ≤ 1, and as ([1/aOR] − 1) × 100% when aOR > 1.

The primary matched-cohort analysis was designed to estimate vaccine effectiveness during the post-vaccination risk period after the child’s in-season vaccination status had been established. Because this approach does not explicitly model changes in vaccination status over calendar time, an additional time-dependent exposure Cox regression analysis was performed in the full eligible cohort as a sensitivity analysis. All children entered follow-up on 4 August 2025 and were followed until influenza onset or 8 February 2026, whichever occurred first. Vaccination status was treated as a time-dependent variable, with person-time classified as unvaccinated before 14 days after the last SIV dose and vaccinated thereafter. Children vaccinated during follow-up could therefore contribute person-time to both exposure states. The model adjusted for the same baseline covariates as in the main analysis, and robust standard errors clustered at the individual level were used to account for repeated records per child after data expansion. aHRs, 95% CIs, and corresponding aVE estimates were calculated as described above.

All analyses were conducted using R software (version 4.4.1), with the MatchIt, cobalt, survival and stats packages. A two-sided *p* value < 0.05 was considered statistically significant.

## 3. Results

### 3.1. Baseline Characteristics Before and After Matching

Before matching, substantial differences were observed between the vaccinated and unvaccinated groups for several variables, including history of pneumococcal vaccination (ASD = 0.429), history of SIV (ASD = 0.624), and missed NIP vaccine doses (ASD = 0.236). After propensity score matching, all covariates were well balanced between the two groups, with ASDs below 0.1, indicating good comparability (Table 1).

### 3.2. Vaccine Effectiveness Against Influenza

As shown in Figure 1, the predefined study period closely matched the temporal distribution of reported influenza activity in Longgang District during the 2025/26 season, covering the onset, rise, peak, and decline of the epidemic. During this period, 1103 influenza cases occurred in the vaccinated group and 1509 in the unvaccinated group. The incidence rates were 28.70 and 40.21 per 100 person-years, respectively. SIV was associated with a significantly lower risk of influenza infection (aHR = 0.71, 95% CI: 0.66–0.77), corresponding to an aVE of 28.82% (95% CI: 23.07–34.14%) (Table 2).

### 3.3. Subgroup Analyses

aVE estimates were generally directionally consistent across most subgroups, although several smaller subgroups yielded imprecise estimates with wide confidence intervals. aVE was similar between children aged 6–35 months (26.78%) and 36–59 months (29.45%) (*p* for interaction = 0.065). By sex, aVE was 25.97% in girls and 31.41% in boys, with no evidence of interaction (*p* for interaction = 0.332). Children without prior SIV showed higher VE than those who had been vaccinated in the previous season (34.28% vs. 20.41%), and this difference was statistically significant (*p* for interaction = 0.016). VE estimates were otherwise generally consistent across subgroups defined by NIP vaccination status, EV71 vaccination, pneumococcal vaccination, and varicella vaccination, with no significant interactions observed.

### 3.4. Analysis by Case Classification and Virus Type

SIV showed similar effectiveness against clinically diagnosed influenza (aVE: 30.18%, 95% CI: 16.71–41.48%) and laboratory-confirmed influenza (aVE: 29.00%, 95% CI: 22.59–34.88%). When stratified by virus type, aVE against influenza A was 29.84% (95% CI: 23.32–35.80%), whereas no significant protection was observed against influenza B or co-infections, likely because of the small number of cases.

### 3.5. Sensitivity Analyses

Sensitivity analyses further supported the robustness of the main findings. In the negative control outcome analysis using varicella infection, no significant association between SIV and varicella infection was observed (adjusted HR = 0.89, 95% CI: 0.39–2.03). However, the small number of varicella events resulted in limited statistical power and a wide confidence interval; therefore, this negative-control analysis does not rule out residual confounding. Consistent with the primary analysis, the multivariable logistic regression model yielded an adjusted OR of 0.70 (95% CI: 0.65–0.76), corresponding to an aVE of 29.58% (95% CI: 23.59–35.11%). In addition, the time-dependent exposure Cox regression model conducted in the full eligible cohort produced a comparable estimate (adjusted HR = 0.70, 95% CI: 0.66–0.75), corresponding to an aVE of 29.42% (95% CI: 24.85–33.71%). Taken together, these findings indicate that the observed protective effect of SIV was robust across different analytical approaches (Table 3).

## 4. Discussion

In this population-based retrospective cohort study based on linked immunization and influenza surveillance data, receipt of egg-based inactivated influenza vaccine was associated with a 28.82% reduction in the risk of influenza among children aged 6–59 months during the 2025/26 influenza season in Shenzhen. Although viral genomic sequencing was not available in this study, multiple lines of evidence suggest that the 2025/26 season in southern China, including Shenzhen, was likely dominated by drifted A(H3N2) subclade K viruses [23,29]. In this context, the generally consistent direction and magnitude of protection across subgroup and sensitivity analyses support the robustness of our main finding despite the observational nature of the study.

The moderate aVE estimated in our study is broadly comparable with reports from some other settings during recent drifted seasons. Our estimate of 28.82% falls within the range of published estimates from young children in Beijing (19.6%, 95% CI: −44.3–55.2%) [23] and Canada (38%, 95% CI: 27–47%) [30]. At the same time, aVE appears lower than some recent estimates reported from France (57.18%) [22], England (74.8%) [24] and the United States (38–41%) [31], These between-study differences should be interpreted cautiously, as they may reflect not only differences in vaccine platforms, but also variation in circulating strains, population characteristics, case definitions, healthcare-seeking behavior, study design, and vaccination timing. Nevertheless, one plausible explanation for the relatively modest aVE observed in our study is the predominance of antigenically drifted A(H3N2) viruses, which may have reduced vaccine-induced protection, particularly for egg-based vaccines. This interpretation is biologically plausible given the known effects of antigenic drift and potential egg-adaptive changes during vaccine production. We also found that aVE against clinically diagnosed influenza (30.18%, 95% CI: 16.71–41.48%) was very similar to that against laboratory-confirmed influenza (29.00%, 95% CI: 22.59–34.88%). This consistency suggests that the inclusion of both case classifications did not materially distort the overall aVE estimate in this surveillance-based study. In addition, the estimated aVE against influenza A was 29.84%, whereas aVE against influenza B and mixed influenza A/B infection was not statistically significant. This was most likely due to the small number of non-A cases rather than evidence of no effect. Because only 96 children received two doses of SIV during the study period, we were also unable to reliably compare the effectiveness of one versus two doses in this age group. The small number of children receiving two doses may partly reflect the relatively high proportion of children with previous-season influenza vaccination history (42.0%) in the vaccinated group, because previously vaccinated children in this age group are generally recommended to receive one dose during the current season.

Local and regional evidence from test-negative design studies in China also provides useful context for our findings. A test-negative case–control study conducted among primary and secondary school students in Shenzhen during the 2023/24 influenza season reported an adjusted VE of 57.06% (95% CI: 48.59–64.13%) [32]. In addition, a Guangdong-based test-negative case–control study conducted during the 2021/22–2023/24 influenza seasons reported an overall VE of 37% (95% CI: 31–43%), with a VE of 32% (95% CI: 19–43%) among children aged 0.5 to <3 years [33]. Although these studies provide relevant local and regional evidence of influenza vaccine protection in children, their estimates were obtained during different influenza seasons and under different virological and epidemiological conditions from those of the present study. Therefore, direct comparison should be made cautiously, particularly because our study was conducted during the 2025/26 season, when antigenically drifted A(H3N2) subclade K viruses predominated.

An additional finding of interest was that aVE was lower among children vaccinated in the previous influenza season than among those without prior-season vaccination. This pattern is broadly consistent with previous studies suggesting that repeated influenza vaccination may attenuate VE in some seasons, particularly during A(H3N2)-predominant epidemics [14]. One possible biological explanation is immune imprinting, whereby early exposure to influenza viruses or vaccines can shape the specificity and breadth of subsequent immune responses. Upon repeated exposure to antigenically related influenza viruses or vaccination, previously established immune responses may be preferentially recalled, potentially limiting the development of broader responses to newly drifted strains. This phenomenon may be particularly relevant during seasons dominated by antigenically drifted A(H3N2) viruses, when the antigenic distance between previously encountered and currently circulating viruses may be greater [14]. However, this remains a plausible mechanism rather than a causal explanation for the lower VE observed among previously vaccinated children in our study. Children with and without prior vaccination may still differ in ways not fully captured by the available covariates, including parental risk perception, healthcare-seeking behavior, timing and completeness of current-season vaccination, and underlying health status. Accordingly, our results should not be interpreted as arguing against annual influenza vaccination; rather, they suggest that vaccine performance may vary according to prior vaccination history in drifted seasons and warrants further investigation.

From a public health perspective, the moderate aVE observed here does not diminish the value of influenza vaccination in young children. Even partial protection may translate into a meaningful reduction in disease burden at the population level in an age group with high susceptibility to infection and substantial healthcare use. Our findings also underscore the importance of continued seasonal VE monitoring, timely characterization of circulating strains, and evaluation of whether broader use of alternative vaccine platforms, such as cell-based or recombinant vaccines, could improve protection in seasons marked by substantial antigenic drift.

However, several limitations should be acknowledged. First, as with all observational studies, residual confounding cannot be completely excluded despite matching, multivariable adjustment, and the negative control outcome analysis. In particular, we lacked information on underlying medical conditions, socioeconomic factors, household exposure patterns, and healthcare-seeking behavior. Second, the primary outcome included both clinically diagnosed and laboratory-confirmed influenza cases. Although this approach increases sensitivity in routine surveillance settings, outcome misclassification, under-ascertainment, and underreporting remain possible and may have biased aVE estimates toward the null. Third, because detailed influenza subtype and viral genomic sequencing data were unavailable, we could not directly confirm the distribution of circulating clades in our study population or estimate clade-specific VE against drifted A(H3N2) variants. Finally, in the primary analysis, the index date was defined according to the final in-season vaccination date, allowing comparison of influenza risk after vaccination status had been fully established and after an immune induction period had elapsed. However, because this approach does not fully model vaccination as a time-varying exposure, some concern about time-related bias remains possible, particularly because children who developed influenza within 14 days after the index date were excluded. Importantly, the time-dependent exposure Cox model in the full eligible cohort produced a highly comparable estimate (aHR = 0.70, 95% CI: 0.66–0.75; aVE = 29.42%, 95% CI: 24.85–33.71%) to that of the primary analysis (aHR = 0.71, 95% CI: 0.66–0.77; aVE = 28.82%, 95% CI: 23.07–34.14%), supporting the robustness of the main result. Although this consistency suggests that the methodological issue was unlikely to have materially affected our conclusions, residual time-related bias cannot be completely excluded.

## 5. Conclusions

In conclusion, SIV provided moderate protection against influenza among children aged 6–59 months during the 2025/26 influenza season in Shenzhen. The reduced effectiveness observed in the context of antigenic drift, together with heterogeneity by prior vaccination history, highlights the need for continued evaluation of vaccine performance and ongoing optimization of influenza vaccination strategies for young children.

## Figures and Tables

**Table 1:** Baseline characteristics of the vaccinated and unvaccinated groups before and after propensity score matching.

Characteristics	Before Propensity Score Matching			After Propensity Score Matching		
	Vaccinated Group N (%) (*n* = 14,291)	Unvaccinated Group N (%) (*n* = 92,901)	ASD	Vaccinated Group N (%) (*n* = 13,239)	Unvaccinated Group N (%) (*n* = 13,205)	ASD
Age (months)			0.123			0.004
6~35	3189 (22.3)	15,964 (17.2)		2993 (22.6)	3005 (22.8)	
36~59	11,102 (77.7)	76,937 (82.8)		10,246 (77.4)	10,200 (77.2)	
Sex			0.023			0.004
Girls	6807 (47.6)	43,178 (46.5)		6330 (47.8)	6339 (48.0)	
Boys	7484 (52.4)	49,723 (53.5)		6909 (52.2)	6866 (52.0)	
Completed the full NIP schedule ^a^			0.019			0.011
Yes	941 (6.6)	5675 (6.1)		833 (6.7)	845 (6.4)	
No	13,350 (93.4)	87,226 (93.9)		12,356 (93.3)	12,360 (93.6)	
Missed any age-eligible NIP dose ^a,b^			0.236			0.009
Yes	279 (2.0)	4844 (5.2)		258 (1.9)	241 (1.8)	
No	14,012 (98.0)	88,057 (94.8)		12,981 (98.1)	12,964 (98.2)	
History of EV71 vaccination ^a^			0.194			0.002
Yes	6973 (48.8)	36,296 (39.1)		6479 (48.9)	6448 (48.8)	
No	7318 (51.2)	56,605 (60.9)		6760 (51.1)	6757 (51.2)	
History of pneumococcal vaccination ^a,c^			0.429			0.002
Yes	9437 (66.0)	42,492 (45.7)		8758 (66.2)	8720 (66.0)	
No	4854 (34.0)	50,409 (54.3)		4481 (33.8)	4485 (34.0)	
History of varicella vaccination ^a^			0.111			<0.001
Yes	11,235 (78.6)	68,855 (74.1)		10,400 (78.6)	10,372 (78.5)	
No	3056 (21.4)	24,046 (25.9)		2839 (21.4)	2833 (21.5)	
History of SIV ^d^			0.624			<0.001
Yes	5916 (41.4)	9917 (10.7)		5559 (42.0)	5545 (42.0)	
No	8375 (58.6)	82,984 (89.3)		7680 (58.0)	7660 (58.0)	
History of reported influenza infection ^d,e^			0.036			<0.001
Yes	542 (3.8)	2881 (3.1)		501 (3.8)	501 (3.8)	
No	13,749 (96.2)	90,020 (96.9)		12,738 (96.2)	12,704 (96.2)	

Abbreviations: ASD, absolute standardized difference; NIP, National Immunization Program (NIP) vaccines are vaccines provided free of charge to children according to national immunization guidelines, targeting diseases of major public health importance—such as hepatitis B, diphtheria, tetanus, pertussis, measles, mumps, rubella, and poliomyelitis—and administered according to a recommended age-specific schedule; EV71, Enterovirus 71; ^a^ Defined on the basis of vaccination records as of 4 August 2025, receipt of non-NIP vaccines that can substitute for NIP vaccines was also considered completion of the corresponding NIP vaccine dose. ^b^ Defined as missing any age-eligible dose of NIP vaccines or their alternative vaccines according to the NIP schedule. ^c^ Including the 13-valent pneumococcal conjugate vaccine (PCV13) and the 23-valent pneumococcal polysaccharide vaccine (PPSV23). ^d^ During the previous influenza season (August 2024 to April 2025). ^a,d^ Vaccination data were obtained from the Shenzhen Immunization Program Information System. ^e^ Based on records from the National Notifiable Infectious Disease Surveillance System.

**Table 2:** Effectiveness of egg-based inactivated seasonal influenza vaccines against influenza in children aged 6–59 months during the 2025/26 influenza season in Shenzhen area, China.

Characteristics	Vaccinated Group (*n* = 13,239)			Unvaccinated Group (*n* = 13,205)			Adjusted HR (95% CI)	Adjusted VE (%) (95% CI)	*p* for Interaction
	No. Influenza Cases	No. p-y	Incidence/100 p-y	No. Influenza Cases	No. p-y	Incidence/100 p-y			
Age (months)									
6~35	252	942.68	26.73	338	928.72	36.39	0.73 (0.62–0.82)	26.78 (13.80–37.80)	0.065
36~59	851	2900.07	29.34	1171	2824.20	41.46	0.71 (0.65–0.77)	29.45 (22.93–35.41)	
Sex									
Girls	545	1849.12	29.47	719	1811.53	39.69	0.74 (0.66–0.83)	25.97 (17.25–33.77)	0.332
Boys	558	1993.63	27.99	790	1941.38	40.69	0.69 (0.62–0.76)	31.41 (23.56–38.46)	
Completed the full NIP schedule ^a^									
Yes	81	234.42	34.55	94	220.99	42.54	0.81 (0.60–1.09)	19.34 (−8.25–40.08)	0.387
No	1022	3608.32	28.32	1415	3531.92	40.06	0.70 (0.65–0.76)	29.48 (23.57–34.93)	
Missed any age–eligible NIP dose ^a,b^									
Yes	24	70.22	34.18	27	66.67	40.50	0.83 (0.48–1.44)	16.97 (−30.56–52.13)	0.540
No	1079	3772.52	28.60	1482	3686.24	40.20	0.71 (0.66–0.77)	29.07 (23.28–34.42)	
History of EV71 vaccination ^a^									
Yes	557	1896.12	29.38	777	1842.74	42.17	0.69 (0.62–0.77)	30.61 (22.63–37.77)	0.520
No	546	1946.58	28.05	732	1910.16	38.32	0.73 (0.65–0.82)	26.92 (18.35–34.58)	
History of pneumococcal vaccination ^a,c^									
Yes	751	2557.04	29.37	1022	2496.75	40.93	0.72 (0.65–0.79)	28.50 (21.44–34.93)	0.854
No	352	1285.70	27.38	487	1256.16	38.77	0.70 (0.61–0.81)	29.62 (19.28–38.64)	
History of varicella vaccination ^a^									
Yes	870	3029.25	28.72	1211	2952.93	41.01	0.70 (0.64–0.76)	30.16 (23.81–35.99)	0.340
No	233	813.50	28.64	298	799.98	37.25	0.77 (0.65–0.91)	23.11 (8.74–35.23)	
History of SIV ^d^									
Yes	484	1662.29	29.12	598	1639.21	36.48	0.80 (0.71–0.90)	20.41 (10.27–29.40)	0.016
No	619	2180.45	28.39	911	2113.70	43.10	0.66 (0.59–0.73)	34.28 (27.22–40.66)	
History of reported influenza infection ^d,e^									
Yes	47	145.50	32.3	55	143.54	38.32	0.83 (0.56–1.22)	17.21 (−18.03–43.94)	0.085
No	1056	3697.25	28.56	1454	3609.37	40.28	0.71 (0.65–0.77)	29.32 (23.50–34.71)	
Influenza case classification									
Clinically diagnosed case	213	3677.39	5.79	293	3537.98	8.28	0.70 (0.59–0.83)	30.18 (16.71–41.48)	
Laboratory–confirmed case	890	3806.73	23.38	1216	3703.04	32.84	0.71 (0.65–0.77)	29.00 (22.59–34.88)	
Influenza case typing									
Influenza A	838	3839.14	21.83	1163	3750.49	31.01	0.70 (0.64–0.77)	29.84 (23.32–35.80)	
Influenza B	11	3685.70	0.3	13	3546.27	0.37	0.83 (0.37–1.85)	17.27 (−45.86–62.94)	
Co-infection influenza A and B	5	3683.94	0.14	4	3544.92	0.11	1.20 (0.32–4.47)	−16.75 (−77.65–67.75)	
Overall	1103	3842.74	28.70	1509	3752.91	40.21	0.71 (0.66–0.77)	28.82 (23.07–34.14)	

Abbreviations: No., number; p-y, person-years; HR, hazard ratio; CI, confidence interval; VE, vaccine effectiveness; NIP vaccines, National Immunization Program (NIP) vaccines are vaccines provided free of charge to children according to national immunization guidelines, targeting diseases of major public health importance—such as hepatitis B, diphtheria, tetanus, pertussis, measles, mumps, rubella, and poliomyelitis—and administered according to a recommended age–specific schedule; EV71, Enterovirus 71; ^a^ Defined on the basis of vaccination records as of 4 August 2025, receipt of non–NIP vaccines that can substitute for NIP vaccines was also considered completion of the corresponding NIP vaccine dose. ^b^ Defined as missing any age–eligible dose of NIP vaccines or their alternative vaccines according to the NIP schedule. ^c^ Including the 13–valent pneumococcal conjugate vaccine (PCV13) and the 23–valent pneumococcal polysaccharide vaccine (PPSV23). ^d^ During the previous influenza season (August 2024 to April 2025). ^a,d^ Vaccination data were obtained from the Shenzhen Immunization Program Information System. ^e^ Based on records from the National Notifiable Infectious Disease Surveillance System.

**Table 3:** Sensitivity analyses using different analytical approaches.

Sensitivity Analysis (Outcome)	β	SE	Wald χ^2^/Z	*p*	Adjusted HR/OR (95% CI)	Adjusted VE (%) (95% CI)
Negative control outcome analysis (varicella) ^a^	−0.12	0.423	0.08	0.778	0.89 (0.39–2.03)	11.25 (−50.73–61.27)
Multivariable logistic regression analysis (influenza) ^b^	−0.35	0.042	70.79	<0.001	0.70 (0.65–0.76)	29.58 (23.59–35.11)
Time-dependent exposure Cox regression analysis (influenza) ^c^	−0.34	0.032	−10.88	<0.001	0.70 (0.66–0.75)	29.42 (24.85–33.71)

Abbreviations: β, regression coefficient; SE, standard error; Wald χ^2^, Wald chi-square statistic; HR, hazard ratio; OR, odds ratio; VE, vaccine effectiveness; CI, confidence interval. ^a^ Varicella was used as a negative control outcome and analyzed using a multivariable Cox regression model for sample after propensity score matching (*n* = 26,444), the number of varicella events was small, resulting in limited statistical power and a wide confidence interval. Therefore, the absence of a statistically significant association should not be interpreted as evidence that residual confounding was completely absent. ^b^ Influenza was analyzed using a multivariable logistic regression model for sample after propensity score matching (*n* = 26,444). ^c^ Influenza was analyzed using a time-dependent exposure Cox regression model for all eligible populations (*n* = 107,192).

## Data Availability

The de-identified data that support the findings of this study are not publicly available because they were derived from routine immunization and infectious disease surveillance systems and are subject to institutional and data protection restrictions. Data may be available from the corresponding author on reasonable request, subject to approval by the Shenzhen Longgang District Center for Disease Control and Prevention and applicable data governance requirements.

## Abbreviations

SIV: seasonal influenza vaccination; VE: vaccine effectiveness; aVE: adjusted vaccine effectiveness; CI: confidence interval; HR: hazard ratio; aHR: adjusted hazard ratio; OR: odds ratio; aOR: adjusted odds ratio; NIP: National Immunization Program; EV71: enterovirus 71; PCV13: 13-valent pneumococcal conjugate vaccine; PPSV23: 23-valent pneumococcal polysaccharide vaccine.
